# The efficiency of the EmERGE platform for medically stable people living with HIV in Portugal

**DOI:** 10.1097/j.pbj.0000000000000191

**Published:** 2022-10-24

**Authors:** Eduard J. Beck, Sundhiya Mandalia, Platonas Yfantopoulos, Christopher I. Jones, Stephen Bremner, Jennifer Whetham, Ana Sofia Cunha, Eugenio Teofilo, Goncalo Rodrigues, Margarida Borges

**Affiliations:** a NPMS-HHC CIC, London, United Kingdom; b Department of Health Services Research and Policy, Faculty of Public Health & Policy, London School of Hygiene & Tropical Medicine, London, United Kingdom; c Department of Primary Care and Public Health, Brighton and Sussex Medical School, Brighton, United Kingdom; d University Hospitals Sussex NHS Foundation Trust, Brighton, United Kingdom; e Hospital Capuchos, Lisbon Centro Hospitalar De Lisboa Central, EPE (HC-CHLC), Lisbon, Portugal.

**Keywords:** cost of HIV services, efficiency EmERGE, mHealth, PLHIV, Portugal

## Abstract

**Background:**

The aim of this study was to calculate the cost-effectiveness of the EmERGE Pathway of Care for medically stable people living with HIV in the Hospital Capuchos, Centro Hospitalar Universitário de Lisboa Central (HC-CHLC). The app enables individuals to receive HIV treatment information and communicate with caregivers.

**Methods:**

This before-and-after study collected the use of services data 1 year before implementation and after implementation of EmERGE from November 1, 2016, to October 30, 2019. Departmental unit costs were calculated and linked to mean use of outpatient services per patient-year (MPPY). Annual costs per patient-year were combined with primary (CD4 count; viral load) and secondary outcomes (PAM-13; PROQOL-HIV).

**Results:**

Five hundred eighty-six EmERGE participants used HIV outpatient services. Annual outpatient visits decreased by 35% from 3.1 MPPY (95% confidence interval [CI]: 3.0–3.3) to 2.0 (95% CI: 1.9–2.1) as did annual costs per patient-year from €301 (95% CI: €288–€316) to €193 (95% CI: €182–€204). Laboratory tests and costs increased by 2%, and radiology investigations decreased by 40% as did costs. Overall annual cost for HIV outpatient services decreased by 5% from €2093 (95% CI: €2071–€2112) to €1984 (95% CI: €1968–€2001); annual outpatient costs decreased from €12,069 (95% CI: €12,047–€12,088) to €11,960 (95% CI: €11,944–€11,977), with 83% of annual cost because of antiretroviral therapy (ART). Primary and secondary outcome measures did not differ substantially between periods.

**Conclusions:**

The EmERGE Pathway produced cost savings after implementation—extended to all people living with HIV additional savings are likely to be produced, which can be used to address other needs. Antiretroviral drugs (ARVs) were the main cost drivers and more expensive in Portugal compared with ARV costs in the other EmERGE sites.

## Background

The aim of this study was to calculate the cost-effectiveness of a new Mobile Health (mHealth) Platform, the *EmERGE Pathway of Care*, developed as part of the *Evaluating mHealth technology in HIV to improve Empowerment and healthcare utilization: Research and Innovation to Generate Evidence for Personalized Care* (EmERGE) Project. EmERGE was implemented in HIV clinics in 5 European countries: Belgium, Croatia, England, Spain, and Portugal.^1^

mHealth is the use of wireless technology to deliver health services and information by mobile communication devices, including mobile phones, tablet computers, and smartphones.^2,3^ mHealth plays an important role in linking care and integrating health services. Improved, integrated health information systems can potentially integrate health services and make them more cost-effective.^4^

EmERGE linked hospital data for medically stable people living with HIV through an application programming interface to a clinical web application. Test results, clinical reviews, and additional messages can be transmitted through a secure socket layer to a messaging server in the cloud. Using a smartphone application (app), patients can securely access their test results, doctors' reviews, or other messages from the cloud server. Similarly, medical appointments and current medications can be viewed, appointments made, and medication reminders set on the app.^1^

The COVID-19 pandemic badly affected Portugal^5^ and reiterated the importance that people living with HIV (PLHIV) or other chronic health need to remain in touch with caregivers, especially during emergencies. Across the world, mHealth has been used increasingly during lockdowns,^6^ including for people living with cancer and mental health disorders,^7,8^ and is likely to continue to be used beyond the COVID-19 pandemic.

The global HIV community recently focused on tracking, monitoring, and evaluating the use, cost, outcome, and impact of health services for PLHIV at individual and country health system levels.^9^ Owing to global access to antiretroviral drugs (ARVs),^10^ the life expectancy of PLHIV now approximates that of people not living with HIV^11^; this will increase the number of PLHIV alive, including those aged 50 years or older.^12^ In high-income countries, non-HIV cancers, cardiovascular disease, and other noncommunicable diseases are the commonest cause of death of PLHIV.^13^ The incidence and prevalence of noncommunicable diseases are also increasing among PLHIV in low-income and middle-income countries.^14^ PLHIV around the world will increasingly need to use HIV and non-HIV health and social services.

A recent review identified a number of communication functions that an “ideal” mHealth app should fulfil.^3^ In addition, the confidentiality and security of personal data collected, transmitted, and stored at either end need to be protected^15^ while the technology needs to be affordable and efficient (Table 1).

**Table 1:** Requirements of a telemedicine system^2,3*,15**^

1. Patient–provider and peer communication
2. Medication and appointment reminders
3. A medication checklist, pill identification function, and list of current and discontinued medicines
4. Laboratory reports (CD4 count, viral load, sexually transmitted infections, glucose, and complete blood count)
5. Pharmacy information
6. Nutrition and fitness trackers
7. Resources, links to social services, substance abuse support, video testimonials, case management
8. Settings (profile picture, password, and alerts)
9. A search functions
10. Affordability and efficiency of the technology*
11. Protecting the confidentiality and security of personal information at rest and in-transit**

Most mHealth studies to date, however, have neither included the costs for developing, implementing, and running these pathways, nor assessed their cost-effectiveness or any cost savings. The need for these studies was recognized long ago,^16,17^ but few have been performed since then.^2,3,18^

The focus of this study was to calculate the annual cost of HIV outpatient services at Hospital Capuchos, Centro Hospitalar Universitário de Lisboa Central, 1 year before and after implementing the EmERGE Pathway. The specific objectives were (1) to calculate the use of outpatient services by EmERGE participants at Hospital Capuchos, (2) to calculate the unit costs of departmental services supporting EmERGE outpatient services, (3) to calculate the annual costs of HIV outpatient services before and after implementation of EmERGE, and (4) to calculate the cost-effectiveness of the implementation of the EmERGE Pathway at Hospital Capuchos, Lisbon, Portugal.

## Methods

### Context

The Centro Hospitalar Universitário de Lisboa Central (CHLC) is the main public healthcare organization in Central Lisbon and comprises 6 hospitals, a number of laboratories, and pharmacies, all under a central administrative structure; they also share laboratory and pharmacy services and specialize in the treatment and management of different diseases. Although HIV services are provided in 3 of the 6 hospitals, Hospital Capuchos (HC-CHLC) manages the largest number of PLHIV at the CHLC and this is where EmERGE participants were recruited (Table 2).

**Table 2:** Inclusion and exclusion criteria for EmERGE participants^19*^

Inclusion criteria: Patients who meet all of the following criteria were eligible for this study:	• Documented HIV infection
	• Aged at least 18 years
	• Able to give informed consent
	• In possession of a smartphone, tablet, or similar technology supporting the mHealth platform
	• Clinically stable on ART. This was defined as receiving ART for at least 1 year and unchanged regimen for at least 3 months, 2 consecutive undetectable viral load measures (<50 copies/ml), no current pregnancy, and without any new WHO clinical stage 2, 3, or 4 events within the previous 12 months*
Exclusion criteria: Patients who met one or more of the following criteria were excluded from this study	• Aged younger than 18 years
	• Pregnant
	• Participating in a clinical trial or receiving an investigational medication
	• Unable to comprehend the patient information sheet
	• Unable to comprehend the instructions for using the mHealth platform
	• Considered for any other reason by their regular physician to be unsuitable for study participation

ART = antiretroviral therapy; mHealth = mobile health.

One doctor managed most of the EmERGE participants, while other doctors, nursing staff, receptionists, and administrators assisted in providing outpatient services at Hospital Capuchos. A microcosting exercise was performed on the outpatient department and other supporting departments. Because very few EmERGE participants used day ward or inpatient services or had surgical procedures, these departments were not costed. The cost of radiological procedures was included in the analyses.

During outpatient consultations, tests could be performed on study participants, ARVs, or other drugs prescribed and all drugs were obtained from the Capuchos Hospital Pharmacy. Although Hospital Capuchos has laboratory facilities, they mainly performed non–HIV-related work; tests for HIV patients were sent for analysis to the Central Laboratory of the CHLC. The results were uploaded online on the hospital information system and reviewed by the requesting doctor, who would discuss these with their patient. As per EmERGE protocol, one visit per year was an electronic “visit” when the physician reviewed the results of blood tests electronically and sent recommendation for future treatment using the EmERGE app; the other routine annual visit was a face-to-face visit.

### Data collected

This before-and-after study collected data on the use, cost, and outcome of HIV services by EmERGE participants before and after implementation of the EmERGE Pathway to estimate its efficiency. This type of study is appropriate for health technology assessments.^20^ Data collected included process information on use of services by individual EmERGE participants one year before and one year after enrolment and the unit cost data of the departments supporting these services. Changes in average annual cost of services and primary or secondary outcome measures before and after EmERGE were analyzed.

### Costing health facilities

The “top-down” or “bottom-up or ingredient-based” costing methods are the main methods to cost a service.^21,22^ The “bottom-up” method defines the type and quantity of input or ingredients used to produce the service output. The “top-down” method is easier to perform and equates the cost of providing services based on the past expenditure. This expenditure is then divided by the number of “products” produced during the period.^21,22^ The method used depends on the availability and detail of the underlying data: Where possible, an ingredient-based approach was applied. The following departments were used by EmERGE participants and included in the microcosting study: HIV Outpatient Clinic, Pharmacy, the Laboratories, and the Radiology Departments at Hospital Capuchos. The overhead departments, which supported these departments, could not be costed individually. The Finance Department in the CHLC apportions an additional percentage to departmental budgets to cover overhead costs, and this percentage was adopted for this study because the timeframe and resources of this study did not permit to microcost all the overhead departments.

### Process data

The use of outpatient services by an individual EmERGE participant in the year before and after EmERGE comprised the individual process data. Because EmERGE participants were medically stable, they predominantly used outpatient services and the focus of the microcosting exercise was on the HIV outpatient and supporting services at HC-CHLC and CHLC.

### Unit costs data

The microcosting study was based on the UNAIDS Costing Manual^21^ and UNAIDS Costing Workbook.^22^ Departmental workload or process data and financial data were collected for each department at Hospital Capuchos involved with EmERGE. The total costs for services in the HIV Outpatient Department were added to the costs of the laboratories, pharmacy, radiology, and overhead costs. The departmental financial data included information on *staff*, *consumables*, *overheads*, *procedures*, and *equipment costs* (SCOPE).^23^ The overhead costs were based on budget weightings provided by the staff of the Financial Department of CHLC. Other data were obtained from a variety of informants working in Hospital Capuchos or the CHLC. As Hospital Capuchos also uses central services, it was difficult to disaggregate some information on the hospital's workload for some central services.

Most laboratory tests were analyzed at the Central Laboratory, and it was not possible to calculate the unit costs of each of the different tests ordered. The different tests were aggregated, and single unit costs estimated for each of the tests combining hematology, biochemistry, and immunology tests performed on EmERGE participants. A similar procedure had to be adopted for the radiology investigations because different types of investigations were performed in different radiology departments within the CHLC.

Each of the CHLC hospitals is supported by overhead services. Security services for Hospital Capuchos and CHLC were outsourced and performed by an external company while another company was responsible for the transportation of blood samples or patients. Overhead services also included the Finance and the Resource Departments, the latter which was responsible for handling the supplies for all departments. The Planning and Control Department was involved in the analyses of process and outcome data of the CLCH. Most of these departments, including the General Management Facilities, Administration, and Human Resources Departments, were located at the Hospital São José, the main administrative center of the CHLC.^23^

### Statistical methods

All participants with a baseline visit contributed to the data and analyses presented. Summary statistics are presented with point estimates and indication of variability and missing data. Linear mixed models were used to calculate difference in averages. Time-weighted changes in the CD4 count and viral load were analyzed over a 2-year period and measured by time point changes during one year before and after EmERGE recruitment.^24^ The MIXED procedure in SAS was used by fitting routine values of CD4 counts and viral loads results as dependent variables. Independent variables included the fixed effects of study visit time points. A covariance matrix was used to model the within-patient errors. Estimates of effects are based on MIXED models and assume any missing data were missing at random. Trends over time are presented as point estimates derived from the models. Viral load data were transformed logarithmically to stabilize their variance.

The mean number of services used per patient-year (MPPY) was calculated using methods used previously,^25-27^ based on the following formula:

Mean per patient-year (MPPY) calculation formula

(1) $$M=\frac{\sum_{i=1}^{\text{n}}\sum_{j=1}^{k}S_{ij}}{\sum_{i=1}^{\text{n}}\sum_{j=1}^{k}\left(t_{ij}-t_{i\left(j-1\right)}\right)}\times 365,$$

where *n* = total number of individuals, *k* = day of censoring, *S_ij_* = use of service by individual i on jth day, *t_ij_* = number of days of follow-up for individual *i*, and *M* = mean of services *S* per patient-year.

The denominator comprised the total duration of follow-up for all patients during a calendar year, one year before when they were entered into this study to one year after study entry. The data were left censored at the one year prebaseline visit. Post-mHealth data were right censored at one year since the baseline visit if patients were still under follow-up at one year, if they had died during follow-up then their date of death, or if they were lost to follow-up, whichever came first. Numerators were calculated by summing the use of outpatient services and calculating the MPPY use of services (Equation 1). Exact Poisson 95% confidence intervals (CIs) were estimated for MPPY based on the distribution of the observed number of outpatient visits divided by the total duration of follow-up for all patients during a calendar year. All statistical analyses were performed using SAS version 9.4 software.^28^

Average annual costs per patient-year (ACPPY) of HIV outpatient services were produced by multiplying MPPY outpatient visit by their respective unit costs. The total annual costs for providing services were obtained by adding the annual costs for outpatient visits, tests, drugs, and procedures performed at the HC-CHLC for EmERGE participants and overheads.^29^ Costings were performed from a societal perspective.

### Primary and secondary outcome measures

Changes in CD4 count and viral load measurements before and after EmERGE were the primary outcome measures. The secondary outcome measures included changes in PAM-13, which is an index of patient activation,^30^ and PROQOL-HIV quality of life measures,^31^ from month 0 (baseline) to month 12 post-mHealth.

### Cost-effectiveness analyses

Incremental cost-effectiveness ratios were calculated based on the annual costs as well as primary and secondary outcome measures before and after the implementation of mHealth using the following formula^32^:

$$\text{ICER}=\frac{\left(\text{Costs A}\;-\;\text{Costs B}\right)\;}{\left(\text{Outcome A}\;-\;\text{Outcome B}\right)}.$$

Because the primary and secondary outcome measures did not change substantially between periods, this study ended up being a cost-minimization study comparing annual costs between periods.

### Out-of-pocket expenditure for EmERGE participants

Information was also collected from study participants on their socioeconomic background, time off work for clinic appointments, return travel time, and costs for clinic appointments.

## Results

Five hundred eighty-six individuals were enrolled and followed up for a year between November 1, 2016, and October 30, 2019; 87% were men, with a mean age at study entry of 42.0 years (95% CI: 39.2–44.8). Of the 551 participants with known employment status, 78% had full-time employment with a median 40 hours of work week (interquartile range [IQR]: 36–44) and a median monthly income of €915 (IQR: €635–€1450). Six percent of participants received additional benefits: 47% of these received pension credits, 47% income support, and 41% “other” benefits. The median monthly income of additional benefits was €222 (IQR: €178–€665).

The median number of sick days 3 months before enrolment was 0 days (IQR: 0 days); 67% of participants did not take a day off work for a clinic visit to attend for appointments with professionals, to attend for blood tests, or to pick up their medication. The median return travel time to their clinic appointment was 2 hours (IQR: 1–3 hours), and the median cost of this return journey was €5 (IQR: €3–€10).

### Estimated unit costs

The unit costs for the EmERGE outpatient and supporting services estimated were calculated as part of the microcosting exercise (Table 3).

**Table 3:** Costs and unit costs of annual outpatient visits, tests, drugs, and investigations performed for EmERGE participants HC-CHLC^23^

Services	Cost/unit cost
Unit cost per EmERGE HIV outpatient visit	€96
Unit cost per EmERGE combined laboratory test	€3
Unit cost per EmERGE radiological investigation	€18
Annual cost of non-ARV pharmacy services for EmERGE patients	€1,135
Annual unit cost of ARVs per EmERGE patient	€9,976

ARV = antiretroviral.

### Annual use and cost of services

Outpatient clinic visits decreased by 35% from 3.1 MPPY (95% CI: 3.0–3.3) to 2.0 MPPY (95% CI: 1.9–2.1; Table 4). Laboratory tests increased by 3% from 246.9 MPPY (95% CI: 244.9–247.5) to 253.1 MPPY (95% CI: 251.9–254.5), whereas radiology investigations decreased by 39% from 2.8 MPPY (95% CI: 2.6–2.9) to 1.7 MPPY (95% CI: 1.6–1.8).

**Table 4:** Mean number of HIV outpatient visits, tests, and average cost per patient-year at HC-CHLC (2017 financial year)

	Before mHealth		After mHealth	
Services	PPY	95% CIs	Mean PPY	95% CIs
Mean outpatient visits	3.1	3.00–3.3	2.0	1.9–2.1
Average costs	€301	€288–€316	€193	€182–€204
Mean laboratory test	246.9	244.9–247.5	253.1	251.9–254.5
Average costs	€741	€735–€743	€759	€756–€764
Mean radiology tests	2.8	2.6–2.9	1.7	1.6–1.8
Average costs	€50	€47–€52	€31	€29–€32
Subtotal average costs	€1,092	€1,070–€1,111	€983	€967–€1,000
Annual pharmacy costs excluding ARVs	€1,001			
Average annual outpatient services costs	€2,093	€2,071–€2,112	€1,984	€1968–€2,001
Annual ARV costs	€9,976			
Total annual costs	€12,069	€12,047–€12,088	€11,960	€11,944–€11,977

ARV = antiretroviral; mHealth = mobile health.

The ACPPY of outpatient visits decreased by 35% from €301 (95% CI: €288–€316) to €193 (95% CI: €182–€204; Table 4). Costs of tests increased by 1%, from €741 ACPPY (95% CI: €735–€743) to €759 ACPPY (95% CI: €756–€764), whereas the radiology cost decreased by 38%, from €50 ACPPY (95% CI: €47–€52) to €31 ACPPY (95% CI: €29–€32).

Overall, average annual cost for outpatient services decreased by 5% from €2,093 (€2,071–€2,112) to €1,984 (€1,968–€2,001). The annual cost of ARVs was €9,976 (Table 3), 83% of annual costs; overall annual costs, including ARVs, decreased by 1% from €12,069 (€12,047–€12,088) to €11,960 (€11,944–€11,977).

### Primary and secondary outcomes before and after mHealth

**Figure 1.**
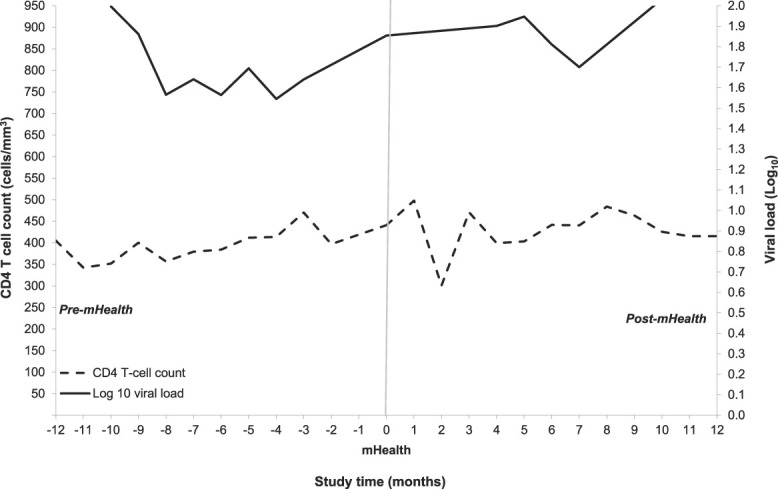
CD4 count and viral load changes before and after EmERGE at HC-CHLC.

The median baseline CD4 count for 91% of the participants was 723 cells/mm^3^ (IQR: 506–944 cells/mm^3^), and CD4 counts did not differ significantly between periods. The median viral load at baseline was 36 copies/mL (IQR: 26–65 copies/mL), while those few who initially had detectable viral loads soon became and remained undetectable (Fig. 1).

Neither EmERGE participants' activation, as measured by PAM-13, nor quality of life indicators based on the PROQOL-HIV changed substantially after EmERGE (Table 5).

**Table 5:** Median and IQR for PAM13 and PROQOL-HIV months 0 and 12, baseline, and post-mHealth at the HC-CHLC

PAM 13	Month 0 (baseline)		Month 12 (after mHealth)	
		Median	IQR	Median	IQR
	70.2	60.6–77.7	67.8	58.1–75.0

PROQOL-HIV	Month 0 (baseline)			Month 12 (after mHealth)		
		n	Median	IQR	n	Median	IQR
Physical health and symptoms	557	83.3	66.7–94.4	172	88.9	72.2–100.0
Body change	550	87.5	68.8–100.0	174	93.8	75.0–100.0
Social relationships	571	100.0	100.0–100.0	174	100.0	100.0–100.0
Intimate relationships	564	75.0	54.2–100.0	174	83.3	58.3–100.0
Stigma	569	37.5	12.5–62.5	174	50.0	12.5–87.5
Emotional distress	564	81.3	56.3–100.0	174	81.3	62.5–100.0
Health concerns	563	50.0	31.3–75.0	174	56.3	31.3–75.0
Treatment impact	550	87.5	77.5–95.0	172	90.0	1.5–95.0

mHealth = mobile health.

## Discussion

The introduction of the EmERGE Pathway has been cost saving, reducing the annual cost of HIV outpatient services by 5%, which was associated with a reduction in outpatient visits. Participants remained clinically stable, and their primary and secondary outcomes did not change substantially. More detailed analyses of the PAM-13 and PROQOL-HIV are presented elsewhere.^33^

The use of the Pathway was well received. Users preferred to use the app away from their work and mostly preferred to do this from home, which empowered users and reduced the risk of disclosure.^34^ Although it was generally agreed that the Pathway provided greater privacy, some privacy concerns remained, especially among black and migrant women. They were anxious that the app could be seen on the phone by friends or family members, who then could ask questions.

The app reduced traveling and waiting times, and virtual sessions were less likely to be interrupted. The virtual calls were on the whole more formal, often focused on discussing results, while face-to-face interactions provided a better opportunity to develop close relationships and facilitate open dialog and negotiations over complex tasks.^34^

The app provided the participants with autonomy because they became less dependent on going to the clinic for routine consultations. The test results function was the most important, and participants, including women and migrants, enjoyed having the EmERGE mHealth app and following the new care pathway.^34^

The introduction of the Pathway also changed the way staff interacted with these participants which might have also resulted in changes in costs. This was unfortunately not quantified during this study, but medical staff indicated that they were able to spend more time on PLHIV with more complex disease.^34^

Overall, the cost of ARVs was the main cost driver for this group of PLHIV, with an annual cost of €9,976 or 83% of overall costs. An additional reduction of outpatient costs could be achieved by switching to quality-assured and affordable generic forms of the ARVs, although the increased use of generic drugs is not without its own issues.^35^ Furthermore, the use of single daily pill regimens can reduce healthcare costs.^36^ Neither of these changes were made in the clinic during the study period. Additional future efficiencies can be achieved, and resources saved by extending the use of the EmERGE Pathway to all PLHIV, including those with more complex HIV disease or people living with other chronic conditions: Savings could be redistributed to address other needs in the clinic.

The most recent Portuguese HIV study on the annual cost of HIV services in Portugal was based on 2006 unit costs and 2008 process data. The estimated annual costs of caring for patients with HIV with CD4 counts ≥500 cells/mm in 2008 was €11,901, of which 81% was due to ARVs.^37^ These historic cost figures are equivalent in purchasing power to €13,975.00 in 2017, and prices were 1.2 times higher than average prices since 2006, based on the European Central Bank consumer price index.^38^

ARVs were found to be the major cost drivers in all 5 EmERGE clinics. Annual expenditure on ARVs of €9,976 was highest in Lisbon compared with annual ARV costs for other sites, which ranged from €4,717 to €8,837 per annum. This difference is accentuated if costs are converted to gross domestic product (purchasing parity prices), an index of a country's wealth^39^: Once transformed, the annual cost of ARVs in Lisbon was US$17,230, compared with US$9,595 to US$11,586 per annum for the other sites.^40^

Costing Hospital Capuchos was a complex exercise, and this complexity limited the use of the bottom-up method and thereby the granularity of the analyses. Although EmERGE participants were followed up at the Hospital Capuchos, some of the laboratories or overhead services that supported the outpatient services used by EmERGE participants were located at other CHLC sites. The financial data collected for Capuchos outpatients included direct costs of staff, consumables, and equipment costs in addition to indirect costs from the laboratories, pharmacy, and overhead costs. To apportion some of the indirect and overhead costs to the outpatient department, this study had to rely on information and weights provided by staff of the Central Finance Department of the CHLC. It was not possible to verify in detail the underlying data that these estimates were based on one of the limitations of this study.

The 6 CHLC hospitals share central services, and it was difficult to get information of which proportion of the total central costs was generated by Hospital Capuchos, especially concerning laboratory tests. All HIV tests were performed in the Central Laboratory, and although it was possible to enumerate the number of tests received from Hospital Capuchos, it was not possible, for instance, to get the total number of HIV tests processed centrally as a proportion of the Central Laboratory workload. Again, approximations had to be made, and the workload of each of the laboratories was aggregated, and a global cost across “all tests” was estimated instead of costing different tests from specific laboratories. These and some of the other challenges comprised limitations of this study; however, enough robust data could be collected for the costing study to produce reliable analyses.

A number of published systematic reviews recently analyzed mHealth interventions. Many highlight the variable effectiveness of mHealth tools depending on different local, national, and international contexts.^16^ The successful implementation of EmERGE in 5 different European countries demonstrated its applicability in different countries and cultural settings.

Some reviews were HIV-specific,^2,3,41^ whereas others reviewed mHealth tools for other chronic diseases.^42,43^ Most studies were performed in high-income countries, but the use of mHealth is increasingly promoted in LMICs.^44-48^ However, conclusions drawn more than 2 decades ago remain relevant today: *Most of the studies analyzed were small scale, short-term, pragmatic evaluations that added little to our knowledge of the costs and benefits that would be expected to result from the introduction of telemedicine services into routine clinical practice*.^16^ This study involved a significant group of participants across different healthcare systems and countries, but only involved medically stable PLHIV, and follow-up was still relatively short.

Protecting the confidentiality and security of the information at rest, on the person's phone or on the institutional server(s), or in-transit is an issue of paramount importance to mHealth.^15^ The range of issues that involves protecting personal health information in such settings has been described elsewhere.^49^ Tools to investigate the level of protection for personal health information at facility, data warehouse, and national levels have also been developed and published.^50^ The extent that they exist and implemented should be regularly reviewed and improved on if implementation has not occurred or been partial.

Although efficiencies were achieved at HC-CHLC, broader implementation of the Pathway to all PLHIV likely leads to greater efficiencies and greater savings. Extending the use of the EmERGE Pathways should be systematically and closely monitored, and their implementation was evaluated and costed.^29^ Funding should be sought from national or international agencies to monitor and evaluate changes in service provision.

Issues raised in this study are, mutatis mutandis, also relevant for people with other chronic diseases, and mHealth is being used for people with chronic diseases.^6^ Funding should go toward development and implementation of similar pathways for other diseases or developing common pathways for people with chronic conditions that are supplemented by disease-specific information. Tools like these would be helpful to deal with other pandemics, including the current COVID-19 pandemic. mHealth contributes to the broader developments of health information system to track the use of country health services by individuals across facilities by linking personal health information over time as part of providing Universal Health Coverage.^51^
